# Privacy-Preserving Handover Optimization Using Federated Learning and LSTM Networks

**DOI:** 10.3390/s24206685

**Published:** 2024-10-17

**Authors:** Wei-Che Chien, Yu Huang, Bo-Yu Chang, Wu-Yuin Hwang

**Affiliations:** 1 Department of Computer Science and Information Engineering, National Dong Hwa University, Hualien City 974301, Taiwan; 611221220@gms.ndhu.edu.tw (Y.H.); 611221240@gms.ndhu.edu.tw (B.-Y.C.); wyhwang@cc.ncu.edu.tw (W.-Y.H.); 2 Graduate Institute of Network Learning Technology, National Central University, Taoyuan City 320317, Taiwan

**Keywords:** federated learning, LSTM, reference signal received power, prediction

## Abstract

The rapid evolution of wireless communication systems necessitates advanced handover mechanisms for seamless connectivity and optimal network performance. Traditional algorithms, like 3GPP Event A3, often struggle with fluctuating signal strengths and dynamic user mobility, leading to frequent handovers and suboptimal resource utilization. This study proposes a novel approach combining Federated Learning (FL) and Long Short-Term Memory (LSTM) networks to predict Reference Signal Received Power (RSRP) and the strongest nearby Reference Signal Received Power (RSRP) signals. Our method leverages FL to ensure data privacy and LSTM to capture temporal dependencies in signal data, enhancing prediction accuracy. We develop a dynamic handover algorithm that adapts to real-time conditions, adjusting thresholds based on predicted signal strengths and historical performance. Extensive experiments with real-world data show our dynamic algorithm significantly outperforms the 3GPP Event A3 algorithm, achieving higher prediction accuracy, reducing unnecessary handovers, and improving overall network performance. In conclusion, this study introduces a data-driven, privacy-preserving approach that leverages advanced machine learning techniques, providing a more efficient and reliable handover mechanism for future wireless networks.

## 1. Introduction

Wireless communication systems have developed rapidly and become an indispensable part of modern society, so privacy protection issues will also receive attention [1]. Seamless connectivity and optimal network performance are crucial, particularly in dense urban environments and high-mobility scenarios. Handover mechanisms, essential for maintaining continuous service as users move between cells, directly impact user experience and network efficiency [2]. Traditional handover algorithms, such as 3GPP Event A3, are widely used. However, signal strength fluctuations and dynamic user mobility are often encountered. For example, if the signal strength at the junction of two base stations is close, the user equipment may switch between the two base stations frequent switching and low resource utilization(i.e., ping-pong effect) [3].

Despite the widespread adoption of algorithms like 3GPP Event A3, these methods face limitations in handling the complexities of modern networks [4]. Issues such as the ping-pong effect, where users frequently switch between cells, and inefficient handover decisions are prevalent. These problems degrade network performance and user experience. Furthermore, the increasing importance of data privacy and security necessitates innovative approaches that protect sensitive information while enhancing handover decisions [5,6]. Addressing these challenges requires intelligent, adaptive algorithms capable of learning from vast amounts of data without compromising privacy.

**Figure 1:**
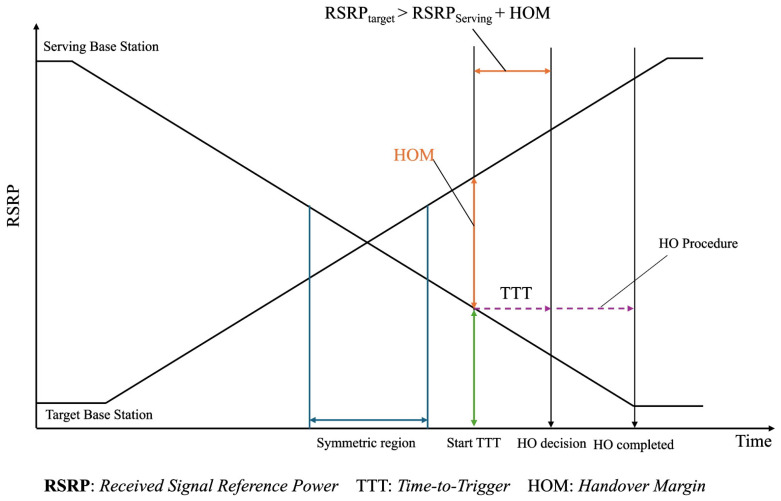
Handover Decision Process with TTT and HOM.

The key parameters for handover control parameters (HCPs) are the time-to-trigger (TTT) and the handover margin (HOM) [7] Figure 1. Proper configuration of these parameters is crucial because it helps mitigate conflicting objectives that may arise during HCP optimization. For instance, setting a high TTT can increase the risk of radio link failure (RLF), while a low TTT setting can elevate the probability of handover ping-pong (HOPP). To address these issues, dynamically adjusting HOM and TTT values is essential. By continuously tuning these parameters based on real-time network conditions, it is possible to balance the trade-offs between RLF and HOPP, thereby ensuring a more stable and efficient network performance [8,9]. Dynamic adjustments of TTT and HOM can significantly contribute to reducing related issues, providing a more adaptable and resilient network that can better handle varying conditions and demands.

LSTM networks can efficiently learn Reference Signal Received Power (RSRP) and the strongest nearby Reference Signal Received Power (NR-RSRP). However, traditional centralized machine learning often overlooks potential privacy issues. To address this, we combined Federated Learning (FL) with our approach, allowing clients to transmit trained model weights during the training process, thereby preventing the leakage of sensitive data. We utilized the FedAvg algorithm for model aggregation, which helps the model avoid local minima, resulting in performance that surpasses centralized LSTM models. Additionally, we conducted experiments on both heterogeneous and homogeneous federated learning to assess the performance differences under varying device computing capabilities and network conditions. Therefore, this study aims to develop a novel approach that combines Federated Learning (FL) and Long Short-Term Memory (LSTM) networks to predict Reference Signal Received Power (RSRP) and the strongest nearby Reference Signal Received Power (RSRP) signals. By leveraging FL, we ensure data privacy through decentralized model training, while LSTM networks capture temporal dependencies and complex patterns in the signal data, improving prediction accuracy. Additionally, we propose a new dynamic handover algorithm that adjusts to real-time network conditions and user mobility patterns, enhancing handover decision-making and reducing unnecessary handovers.

## 2. Related

### 2.1. Traditional Handover Algorithm and Intelligent Handover Algorithms

Traditional handover algorithms, such as the 3GPP Event A3, have been widely adopted in wireless communication systems. These algorithms primarily rely on signal strength measurements, such as Reference Signal Received Power (RSRP), to make handover decisions. Studies have shown that the Event A3 algorithm, while robust, can lead to frequent handovers and increased signaling overhead, particularly in dense urban areas and high-mobility situations [10]. In a highly dynamic environment, traditional handover algorithms may struggle when the signal strengths at the junction of two base stations are nearly identical, leading to frequent handovers, known as the ping-pong effect. To address this issue, our research leverages machine learning to predict future signal strengths and uses these predictions as thresholds in the handover algorithm. This dynamic adjustment aims to reduce the occurrence of the ping-pong effect, resulting in more stable connections for the user equipment.

In recent years, machine learning (ML) techniques have been increasingly applied to enhance handover decisions. Supervised learning models, such as Support Vector Machines (SVM) and Decision Trees, have been used to predict handover triggers based on historical data [11,12]. These approaches have demonstrated improvements in handover accuracy and a reduction in unnecessary handovers. However, they often require large labeled datasets and may not generalize well to unseen environments.

### 2.2. Deep Learning Approaches and Federated Learning for Handover Management

Deep learning techniques, particularly Long Short-Term Memory (LSTM) networks, have been extensively explored for improving handover management in wireless communication systems. LSTM networks are well-suited for capturing temporal dependencies in signal strength data, making them ideal for predicting handover events. For instance, ref.  [13] proposed an LSTM-based handover prediction model that significantly improved handover prediction accuracy in mobile networks. Their study demonstrated that LSTM networks could effectively learn from historical signal data to predict future handover events, thereby reducing the likelihood of missed or unnecessary handovers. Similarly, ref. [14] demonstrated the application of deep learning for 5G handover prediction, highlighting its effectiveness in reducing the ping-pong effect and increasing handover success rates. Their research emphasized the ability of deep learning models to adapt to the complex and dynamic nature of 5G networks, where traditional handover algorithms often fall short. Ref. [15] demonstrated that LSTM networks significantly enhance handover prediction accuracy. Their LSTM-based handover prediction model effectively learns from historical signal data to forecast future handover events, reducing the likelihood of missed or unnecessary handovers. Ref. [16] discusses the implementation of a novel handover management system leveraging Software-Defined Networking (SDN) and Multiplicative Long Short-Term Memory (mLSTM) networks within 5G environments. Despite these advancements, deep learning models often rely on centralized data collection, which raises concerns about data privacy and security. The centralization of data can lead to potential breaches of sensitive user information, making it crucial to explore decentralized approaches.

However, challenges remain, such as the need for high-quality data and the difficulty in generalizing models to unseen environments. To address these, federated learning ensures privacy, lightweight models reduce computational demands, and continuous training improves adaptability across diverse network conditions.

### 2.3. Federated Learning for Handover Management

Federated Learning (FL) is an emerging technique that addresses the privacy issues associated with centralized data collection by enabling decentralized model training. Ref. [17] explores the application of federated learning to enhance handover performance in millimeter-wave (mmWave) vehicular networks. Millimeter-wave communication, characterized by high frequency and large bandwidth, offers significant advantages for vehicular networks. However, it also presents challenges such as rapid signal attenuation and poor penetration, leading to frequent handovers to maintain the quality of service (QoS) for mobile users. Ref. [18] introduces a federated learning framework tailored for 6G networks. It addresses the challenges of heterogeneous data and model aggregation in massive IoT environments, enhancing communication efficiency and model robustness.

In summary, the integration of deep learning and federated learning presents a promising avenue for advancing handover management in next-generation wireless networks. While deep learning offers powerful tools for extracting insights from complex data, federated learning ensures that these insights can be obtained without compromising user privacy. This dual approach is particularly crucial in the era of 5G and beyond, where the volume and variety of data are expected to grow exponentially. The ongoing research in this area continues to push the boundaries of what is possible, paving the way for more intelligent, efficient, and secure handover management solutions.

## 3. Method

### 3.1. Proposed Structure

**Figure 2:**
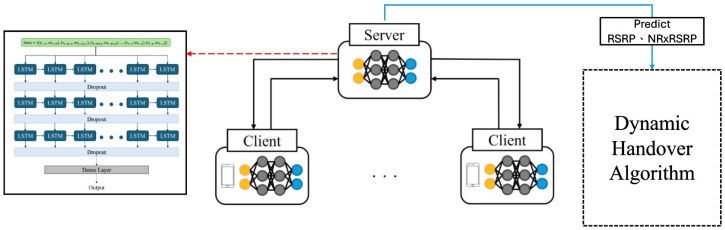
Proposed system structure.

Our goal is to use the LSTM model to learn the spatio-temporal information of Reference Signal Received Power (RSRP) and nearest Reference Signal Received Power (NR-RSRP), thereby reducing unnecessary handovers. Figure 2 shows the system scheme we proposed.

In our research we employ the Federated Long Short-Term Memory (F-LSTM) model to predict future RSRP values and the RSRP of neighboring cells, which are then used to optimize handover events. The F-LSTM model is composed of a global model and multiple local models trained by various clients. Each client trains its LSTM model locally using its own data, capturing temporal dependencies in signal strength without sharing raw data. After local training, only the model weights are sent to a central server, where the FedAvg algorithm aggregates these updates into a global model. This global model is then redistributed to the clients for further training. By integrating Federated Learning and LSTM, our system enhances handover prediction while preserving privacy, allowing for real-time handover decisions through dynamic threshold adjustments based on predicted RSRP values. This approach effectively reduces unnecessary handovers and mitigates the ping-pong effect. In the revised manuscript, we will include more details on the LSTM architecture, data flow, and model synchronization to provide a clearer understanding of the implementation.

#### Detailed Working of the Proposed Structure

- **Data Collection and Preprocessing**
  Clients collect RSRP and NR-RSRP data over time, capturing the dynamic changes in signal strength. The data are preprocessed to handle missing values and normalize the range, ensuring consistency across different clients.
- **Local Model Training**
  Each client uses an LSTM network to train on its local dataset. The LSTM model captures the temporal dependencies in the RSRP data, learning the patterns that indicate signal strength variations. Dropout layers are included to prevent overfitting and enhance generalization.
- **Federated Learning Process**
  After local training, each client sends its model updates (e.g., weights) to a central server. The server aggregates these updates to form a global model, which benefits from the diverse data distributions of all clients.
- **Global Model Distribution**
  The updated global model is then redistributed to the clients. This iterative process ensures that the global model continuously improves and adapts to new data without requiring centralized data storage.
- **Handover Optimization**
  The global F-LSTM model predicts the future RSRP and NR-RSRP for each client. The predictions are fed into a dynamic handover algorithm, which adjusts handover thresholds based on predicted signal strengths and patterns. The algorithm reduces unnecessary handovers by considering both the predicted future RSRP and the current signal environment, thus enhancing overall network performance.

### 3.2. F-LSTM Model

**Figure 3:**
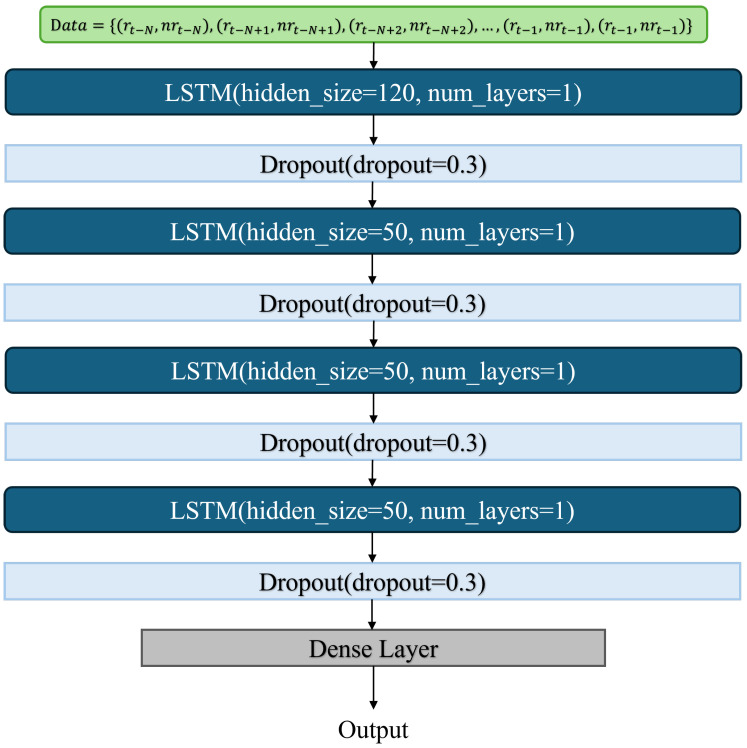
LSTM Model Structure.

In F-LSTM, we use 10 clients for federated learning training. Figure 3 shows the architecture of the LSTM model in F-LSTM, with an input data time interval of 10 ms (the same as the time interval of each data in the dataset). The model consists of four LSTM layers with hidden feature output sizes of [120, 50, 50, 50]. To prevent overfitting, we apply Dropout regularization after the output of each LSTM layer. Finally, a dense layer is used for the output.

To reduce the occurrence of the ping-pong effect, we use the LSTM model to capture the temporal information of RSRP and NR-RSRP. Figure 3 shows the model architecture we use. We input the historical RSRP and NR-RSRP from the dataset into the model, enabling it to predict future RSRP and NR-RSRP values. We represent the historical RSRP and NR-RSRP information from the dataset as follows:

(1) $$D=\left\{(RSRP_{i},NRRSRP_{i})|i=1,2,\ldots ,n\right\},$$

In our dataset, NR-RSRP values have missing entries. We utilize interpolation methods to fill these missing values as follows:

(2) $$y_{i}=y_{i-1}+\left(x_{i}-x_{i-1}\right)/\left(x_{i+1}-x_{i-1}*\left(y_{i+1}-y_{i-1}\right)\right),$$

After filling the missing data, we normalize the entire dataset to a range of 0 to 1. Upon completing all data preprocessing steps, the dataset is ready to be used as input for federated learning training.

In the framework of federated learning, we provide a system model that allows clients to train the model using their own data. After completing the local training, clients return the model weights to the system for aggregation.

We employ the FedAvg [19] architecture, with the model aggregation formula as follows:

(3) $$\forall k,w_{t+1}^{k}\leftarrow w_{t}-\eta *g_{k}$$

For each client *k*, the updated weight $w_{t+1}^{k}$ at time t+1 is computed by subtracting the product of the learning rate η and the gradient $g_{k}$ from the current global weight $w_{t}$. This formula ensures that each client’s model weights are adjusted based on their local gradients, contributing to the overall aggregation process in the federated learning system.

In the t+1 round of global model training on the server, the global weights $w_{t+1}$ are updated by computing a weighted average of the locally trained weights from each client. This approach, as depicted in the formula below, helps to reduce the communication cost associated with training the model:

(4) $$w_{t+1}=\sum_{k=1}^{K}n_{k}/n*w_{t+1}^{k}$$

where *K* represents the total number of clients, $n_{k}$ denotes the number of data samples held by client *k*, and *n* is the total number of data samples across all clients. This weighted averaging ensures that clients with more data have a proportionally greater influence on the global model update.

After the global aggregation is completed, the trained system model can be used to predict future RSRP and NR-RSRP values. These predicted values serve as inputs for the dynamic handover algorithm and act as reference values for triggering handovers.

### 3.3. Dynamic Handover Algorithm

The Algorithm 1 is designed to optimize handover decisions in wireless communication systems by utilizing predicted signal strengths. It aims to improve the overall network performance by reducing unnecessary handovers. The algorithm takes as input the test set of handover triggers (ho_trig_test), predicted Reference Signal Received Power (RSRP) values (predicted_rsrp), predicted nearest Reference Signal Received Power (NR-RSRP) values (predicted_nrxrsrp), and the Mean Absolute Error of the RSRP predictions (mae_rsrp). A key parameter in this algorithm is the continuity_threshold, set to 3, which determines the minimum count for a valid handover decision.
**Algorithm** **1** Dynamic Adjustment Algorithm**Input:** ho_trig_test, predicted_rsrp, predicted_nrxrsrp, mae_rsrp**Output:** optimized_handover_predictions, handover_trigger_points,               event_a3_handover_points**Initialize:** handover_trigger_points←[],                  optimized_handover_predictions←zeros_like(ho_trig_test),                  continuity_count←0,                  base_dynamic_threshold←mae_rsrp,                  ping_pong_effects←0,                  time_window←100**for** *i* **in** **range** (len(val_df)−time_window) **do**   window_data←val_df[i:i+time_window]   **if** $(window\text{\_}data{[}^{\prime }CellID^{\prime }]\left[0\right]\neq window\text{\_}data{[}^{\prime }CellID^{\prime }]$   **and** $window\text{\_}data{[}^{\prime }CellID^{\prime }]\left[0\right]=window\text{\_}data{[}^{\prime }CellID^{\prime }]\left[-2\right]$   **and** $window\text{\_}data{[}^{\prime }CellID^{\prime }]\left[-1\right]=window\text{\_}data{[}^{\prime }CellID^{\prime }]\left[1\right])$ **then**       ping_pong_effects←ping_pong_effects+1   **end if****end for**adjusted_TTT_threshold←base_TTT_threshold+ping_pong_effects**for** each (pred_rsrp,pred_nrxrsrp) in zip(predicted_rsrp, predicted_nrxrsrp) with index *i* **do**   **if** i>0 **then**       prev_avg_rsrp←mean(predicted_rsrp[max(i−3,0)toi])       prev_avg_nrxrsrp←mean(predicted_nrxrsrp[max(i−3,0)toi])   **else**       prev_avg_rsrp←pred_rsrp       prev_avg_nrxrsrp←pred_nrxrsrp   **end if**   dynamic_threshold←base_dynamic_threshold×(1+0.5×abs(prev_avg_rsrp−pred_rsrp)/mae_rsrp)   **if** pred_rsrp>prev_avg_rsrp−dynamic_threshold and pred_nrxrsrp>prev_avg_nrxrsrp **then**     continuity_count←continuity_count+1     **if** continuity_count≥continuity_threshold **then**        optimized_handover_predictions[i]←1     **end if**   **else**      continuity_count←0   **end if****end for****Return:** optimized_handover_predictions, handover_trigger_points,              event_a3_handover_points

The algorithm initializes several variables: handover_trigger_points as an empty list to store the points where handovers are triggered, optimized_handover_predictions as an array of zeros with the same shape as ho_trig_test, and continuity_count to track the count of consecutive valid handover conditions. Additionally, base_TTT_threshold is set to 10, representing the base Time to Trigger threshold, and base_dynamic_threshold is set to the mean absolute error of RSRP predictions. Other variables initialized include newalcount set to 0, ping_pong_effects set to 0, and time_window set to 100, which defines the size of the analysis window.

The next step will start calculating the number of ping-pong effects, which occur when a mobile device rapidly switches back and forth between two base stations. This is conducted by sliding a window of size time_window over the dataset and checking if the first cell in the window is different from the last cell, but matches the second-to-last cell, and if the last cell matches the second cell. If these conditions are met, the ping-pong effect counter is incremented. After counting the ping-pong effects, the algorithm adjusts the Time-To-Trigger threshold by increasing it based on the number of detected ping-pong effects. This adjustment aims to make the handover decision process more stringent in environments prone to frequent, unnecessary handovers, thereby enhancing network stability and reducing rapid, back-and-forth handovers.

The core of the algorithm is a loop that iterates over each pair of predicted RSRP and NR-RSRP values with their respective index i. For each iteration, the algorithm calculates the previous average RSRP and NR-RSRP. If i is greater than 0, it computes these averages over the past three values or fewer if i is less than 3. If i is 0, the previous averages are set to the current predicted values.

Next, the algorithm adjusts the dynamic_threshold, which is computed based on the base_dynamic_threshold and the deviation of the current predicted RSRP from the previous average RSRP, normalized by mae_rsrp. The handover conditions are then evaluated: if the current predicted RSRP exceeds the previous average RSRP minus the dynamic threshold and the current predicted NR-RSRP is greater than the previous average NR-RSRP, the continuity_count is incremented. If the continuity_count meets or exceeds the adjusted TTT threshold, optimized_handover_predictions[i] is set to 1. If the conditions are not met, the continuity_count is reset to 0.

This loop continues until all predicted values are processed. In summary, the Algorithm 1 leverages real-time signal strength predictions to make more informed and optimized handover decisions, effectively minimizing unnecessary handovers and enhancing the overall efficiency of the wireless network.

**Figure 4:**
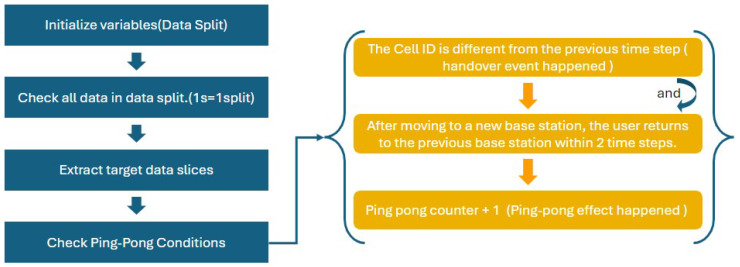
Explanation of the Process for Checking Ping-Pong Conditions.

By utilizing Figure 4, the identification and calculation of ping-pong effects during handovers in mobile networks can be achieved. Ping-pong effects occur when a mobile device rapidly switches back and forth between two base stations within a short period. This can be further validated to ensure that the algorithm meets expected performance standards.

**Figure 5:**
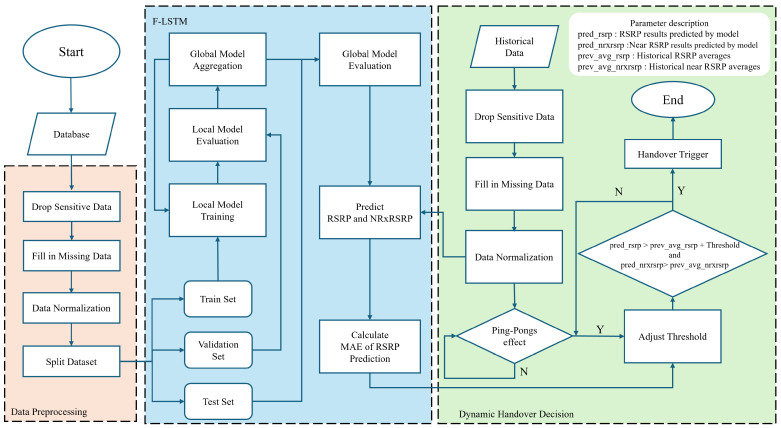
Our integral system flowchart.

Figure 5 is divided into three parts, they are Data Preprocessing, F-LSTM and Dynamic Handover Decision. Data Preprocessing prepares the dataset by handling missing data, normalizing values, and removing sensitive information. F-LSTM explains, trains and aggregates local models in a federated learning framework, predicting RSRP and NRxRSRP values. The Dynamic Handover Decision statement uses predicted and historical data to make intelligent, real-time handover decisions, adjusting thresholds based on detected network conditions like the ping-pong effect, and the upper right corner is annotations for relevant parameters.

## 4. Results

### 4.1. Dataset

The dataset used in our experiment is sourced from [20], this dataset offers extensive multi-source data suitable for predicting cellular traffic, providing crucial insights for developing and refining handover management algorithms. The dataset’s comprehensive nature and real-world applicability make it an excellent foundation for validating the effectiveness of our proposed dynamic handover algorithm.

This dataset was collected from the real 5G production network of a major Irish telecom operator, covering two mobility modes: static and driving. It includes various application scenarios, such as video streaming and file downloads. This dataset is particularly suitable for handover prediction and signal strength forecasting in mobile communication networks, as it contains rich key performance indicators, such as RSRP, Signal-to-Noise Ratio (SNR), and geographical information like UE mobility speed, longitude, and latitude, as shown in Table 1.

The dataset is categorized into two modes: static and dynamic. Given that this study focuses on the 5G handover process, we specifically utilized the dataset collected under dynamic conditions. In the dynamic data collection, mobile devices were placed in vehicles moving through urban and suburban environments, capturing real-world mobility scenarios that are crucial for handover analysis. The velocity of the clients during the dynamic collection ranged from 0 to 57 kph, covering a range of typical vehicular speeds in daily traffic. This range provides a comprehensive basis for studying the impact of mobility on handover performance in 5G networks, as fluctuations in speed can significantly affect signal strength and the frequency of handover events.

**Figure 6:**
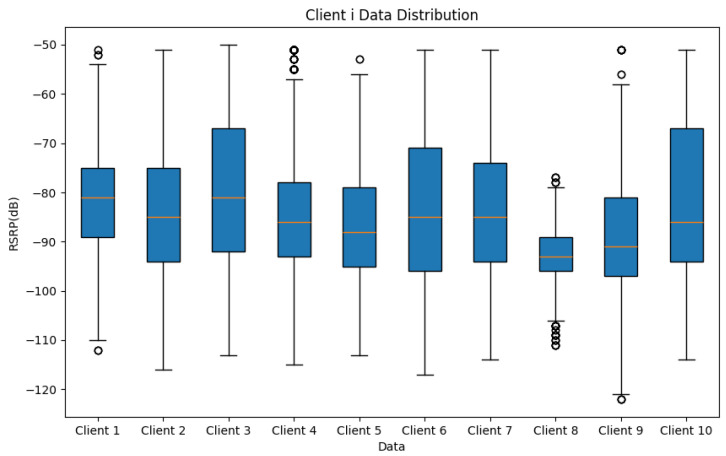
Clients’ Data Distribution, the circles in this figure are the outliers in each clients.

We utilized the dataset and allocated the data to 10 clients, ensuring that no training data were duplicated. Figure 6 illustrates the distribution of RSRP training data in each client, highlighting the diversity of the heterogeneous data. This diversity is crucial for ensuring the model’s reliability.

### 4.2. Model Comparison

This section uses heterogeneous federated learning, homogeneous federated learning and the centralized LSTM model for comparative experiments.

- Heterogeneous federated learning: In our federated learning framework, we use Flower [21]. The server is equipped with an Intel i5-13500 CPU and an RTX 4060 GPU to perform model aggregation, while Raspberry Pi 4 Model B (Raspberry Pi Trading Ltd., Cambridge, UK) are utilized as clients for model training.
- Homogeneous federated learning: We utilize a computer equipped with an Intel i5-13500 CPU and an RTX 4060 GPU for model training and aggregation. For the simulation of federated learning frameworks, we employ the Virtual Client Engine provided by Flower [21].
- Centralized LSTM model [14]: We utilize a computer equipped with an Intel i5-13500 CPU and an RTX 4060 GPU, along with PyTorch, for model training. Long Short-Term Memory (LSTM) networks are primarily employed to address issues related to memory retention and gradient vanishing in long-term sequence training.

### 4.3. Model Performance

**Figure 7:**
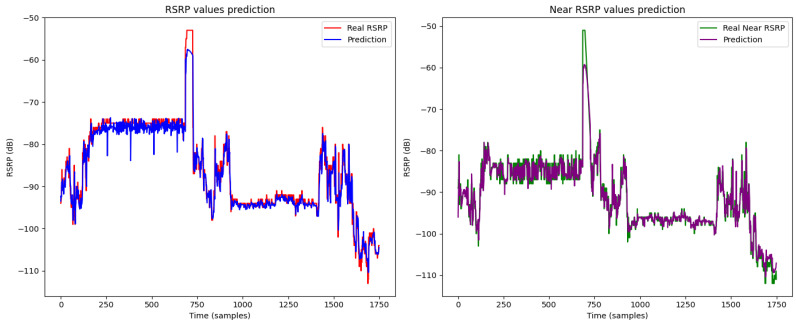
Prediction result of heterogeneous federated learning.

**Figure 8:**
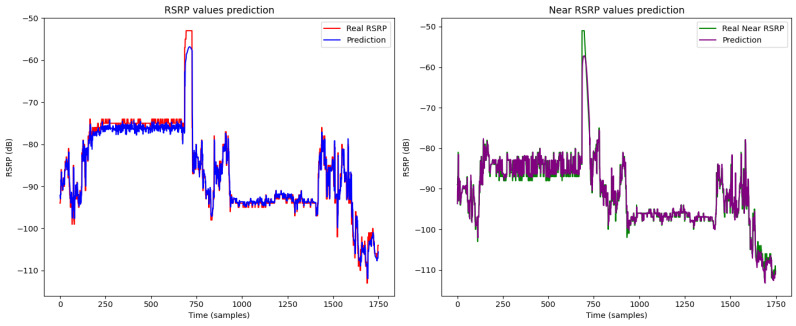
Prediction result of homogeneous federated learning.

**Figure 9:**
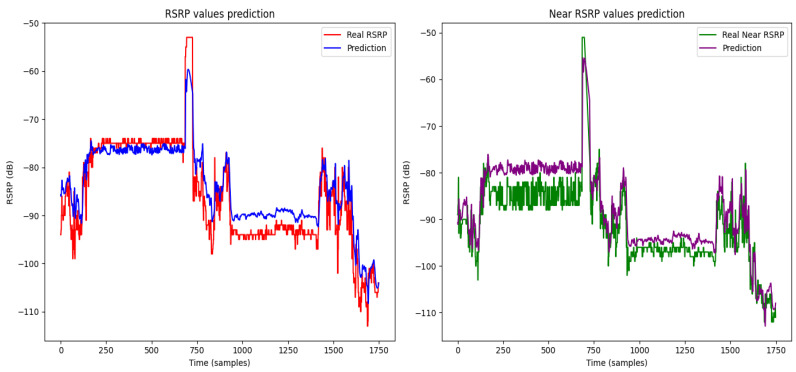
Prediction result of centralized LSTM model.

The figures illustrate the prediction results of heterogeneous federated learning Figure 7, homogeneous federated learning Figure 8, and the centralized LSTM model Figure 9.

In Figure 7, the RSRP and near RSRP predictions using heterogeneous federated learning show a strong alignment with the actual values. This indicates that the model is highly effective in capturing the variations in signal strength and nearby cell signals under diverse conditions.

Figure 8 presents the results for homogeneous federated learning. The RSRP predictions remain closely matched with the actual values, demonstrating the model’s robustness in preserving data privacy while maintaining prediction accuracy. The near RSRP values also follow the actual data trends well, highlighting the model’s capability in a more homogenous setup.

Figure 9 showcases the centralized LSTM model’s performance. The RSRP and near RSRP predictions again align well with the real values, indicating the centralized approach’s effectiveness. However, the variations captured might be slightly less pronounced compared to federated learning methods, suggesting a potential trade-off between centralization and adaptability to diverse data sources.

Overall, these figures demonstrate the effectiveness of our dynamic algorithm across different learning paradigms. The results underscore the model’s robustness in predicting signal strength and nearby cell signals, adapting well to various network conditions simulated by the different datasets. This comprehensive analysis supports the potential of our approach to significantly enhance handover prediction and network performance.

### 4.4. Evaluation Metrics

To evaluate the model’s predictive performance, several metrics should be selected. The model predictions represent $\hat{y_{ij}}$, and include predicted RSRP and predicted NR-RSRP. The true value is represented as $y_{ij}$. Mean Absolute Error (MAE) represents the average absolute difference between the true values and the predicted values.

(5) $$MAE=\frac{1}{nm}\sum_{i=1}^{n}\sum_{j=1}^{m}|y_{ij}-{\hat{y}}_{ij}|$$

Mean Squared Error (MSE) calculates the average of the squared differences between the predicted values and the true values. The closer the model’s predictions are to the true values, the smaller the MSE.

(6) $$MSE=\frac{1}{nm}\sum_{i=1}^{n}\sum_{j=1}^{m}{\left(y_{ij}-{\hat{y}}_{ij}\right)}^{2}$$

Root Mean Squared Error (RMSE) is the square root of the mean squared differences between the predicted values and the true values. Similar to MSE, the closer the model’s predictions are to the true values, the smaller the RMSE.

(7) $$RMSE=\sqrt{\frac{1}{nm}\sum_{i=1}^{n}\sum_{j=1}^{m}{\left(y_{ij}-{\hat{y}}_{ij}\right)}^{2}}$$

Table 2 presents a comparison of model prediction performance across three different approaches: Heterogeneous Federated Learning Hete_FL, Homogeneous Federated Learning Homo_FL, and Centralized LSTM Model Cen_LSTM. The performance metrics considered are Mean Absolute Error (MAE), Mean Squared Error MSE, and Root Mean Squared Error (RMSE). The results highlight several advantages: The Hete_FL approach demonstrates superior accuracy in predictions with the lowest MAE of 3.5021, the smallest overall squared error with an MSE of 5.2394, and the least deviation from the actual values with an RMSE of 2.2883. Similarly, the Homo_FL approach shows significantly better performance compared to the centralized LSTM model, with an MAE of 3.3223, an MSE of 4.6171, and an RMSE of 2.1476, indicating more accurate predictions and better prediction accuracy. Overall, the table indicates that federated learning approaches, both heterogeneous and homogeneous, outperform the centralized LSTM model, with Hete_FL standing out as the most effective method, delivering the highest accuracy and lowest error rates across all metrics. First, the three methods are categorized into federated learning and centralized machine learning. For model aggregation in federated learning, we employ the FedAvg algorithm, which helps guide model training away from local minima. As a result, federated learning tends to outperform centralized machine learning. In the case of heterogeneous federated learning, where different devices are connected via the network, model aggregation can be affected by factors such as network latency, connection stability, or variations in device computing capabilities. Consequently, across all three performance metrics, homogeneous federated learning outperforms heterogeneous federated learning.

### 4.5. Dynamic Handover Algorithm Performance

Table 3 presents the model prediction accuracy across different datasets and learning methods, highlighting the exceptional performance of the dynamic algorithm.

This study employs three different datasets—HSPA+/LTE/5G, LTE/5G, and 5G—to simulate various network conditions and evaluate the performance of different learning methods: Heterogeneous Federated Learning (Hete_FL), Homogeneous Federated Learning (Homo_FL), and a Centralized LSTM Model (Cen_LSTM). The results underscore the significant success of the dynamic algorithm in adapting to these diverse conditions.

Heterogeneous Federated Learning (Hete_FL) achieved the highest accuracy across all datasets, especially in the 5G dataset with an accuracy of 0.9460. This indicates that (Hete_FL) is highly effective in handling diverse and heterogeneous data sources, which is essential in real-world network environments where data can vary significantly.

Homogeneous Federated Learning (Homo_FL) also demonstrated strong performance, particularly in the 5G dataset with an accuracy of 0.9901. This method excels in environments where data sources are more uniform, showcasing its robustness and reliability in such scenarios.

The centralized LSTM Model (Cen_LSTM), while slightly lower in accuracy compared to the federated learning approaches, still delivered impressive results. It performed particularly well in the LTE/5G dataset with an accuracy of 0.9647, highlighting its capability to maintain high performance in less heterogeneous conditions.

As shown in the Table 3, Hete_FL and Homo_FL demonstrated similarly high performance in all tested network environments, including HSPA+/LTE/5G, LTE/5G, and 5G. The minimal differences between the two methods indicate that both are robust and effective in handling different network conditions. The slight advantage of Hete_FL in heterogeneous environments may be attributed to its ability to better manage changes in profile distribution. However, the small performance gap shows that Homo_FL can adapt well even in cases where the data distribution may not be completely uniform.

This similarity in performance highlights the flexibility and advantages of federated learning, regardless of whether the data environment is more homogeneous or heterogeneous. The centralized LSTM model, while still effective, exhibits slightly lower performance, especially in more advanced network scenarios such as 5G, possibly due to its centralized nature, which may limit its ability to capture the inherent limitations of distributed data sources and diversity capabilities.

**Figure 10:**
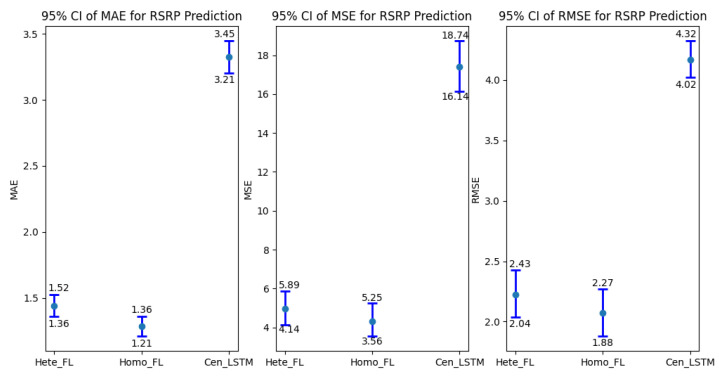
95% Confidence Interval for RSRP Prediction.

In Figure 10 presents the 95% confidence intervals (CI) for three key performance indicators—MAE, MSE, and RMSE —across three models: Hete_FL, Homo_FL, and Cen_LSTM. The results clearly demonstrate that Hete_FL outperforms the other models on all metrics, exhibiting the lowest MAE, MSE, and RMSE, which indicates superior accuracy and reduced error dispersion. The tight confidence intervals for Hete_FL further enhance the credibility of these findings, showcasing the model’s reliability and consistency in predicting RSRP values. In contrast, Cen_LSTM displays wider confidence intervals, reflecting greater variability and lower prediction accuracy, which underscores the advantages of federated learning methods, particularly in heterogeneous environments. The use of confidence intervals in this analysis highlights the stability and practical effectiveness of the Hete_FL model proposed in this study.

**Figure 11:**
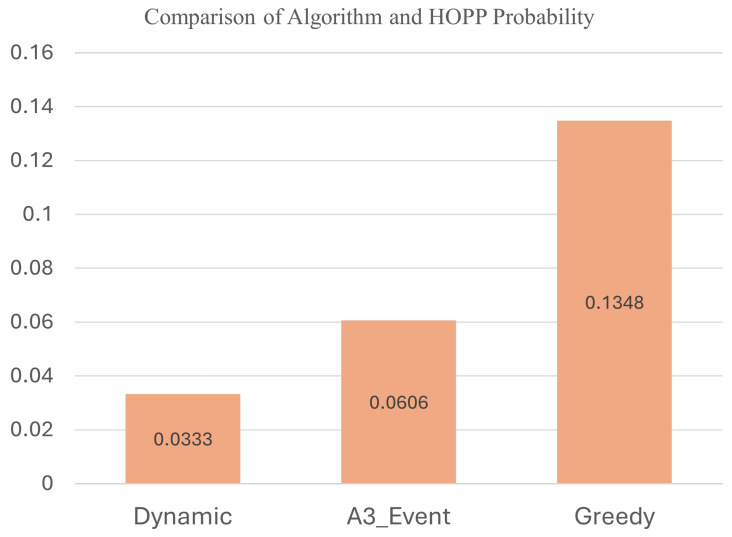
Comparison of Algorithm and HOPP Probability.

In Figure 11 illustrates the Handover Probability (HOPP) for three different algorithms: Dynamic, A3_Event, and Greedy. The Dynamic algorithm exhibited the lowest HOPP probability at 0.0333, demonstrating its effectiveness in reducing unnecessary handovers. This indicates a more conservative approach, resulting in fewer handovers and a more stable connection. In contrast, the A3_Event algorithm showed a HOPP probability of 0.0606, and the Greedy algorithm had the highest HOPP probability at 0.1348. The lower HOPP probability of the Dynamic algorithm highlights its success in optimizing handovers, making it particularly suitable for environments with varying signal conditions and mobility patterns. This result underscores the Dynamic algorithm’s ability to maintain a balance between handover frequency and connection quality, enhancing overall network performance.

The training time refers to the duration of the model training process. The execution time is the duration from loading the trained model to the completion of the prediction, while the prediction time is the time taken for the model to forecast future RSRP values. As shown in Table 4, the differences in execution and prediction times among the three model architectures are minimal. The centralized LSTM model has the shortest time since it does not involve model aggregation and transmission across multiple devices. However, in terms of training time, heterogeneous federated learning takes longer due to the clients’ computational capabilities and the overhead of weight transmission. In our study, the proposed model architecture achieves an execution and prediction time of less than 1 s, with the LSTM prediction time step set to 200 (each data interval is 10 ms). This performance is sufficient to meet most real-time application requirements in practical environments, demonstrating its feasibility.

## 5. Discussion

Our study demonstrates the effectiveness of the dynamic handover algorithm in reducing unnecessary handovers while maintaining network performance. The Dynamic algorithm significantly outperforms traditional methods, such as the A3_Event and Greedy algorithms, by adapting to real-time network conditions. This adaptability leads to a substantial decrease in the probability of HOPP events, which are a common issue in mobile networks.

Our approach leverages the predictive power of LSTM models to forecast RSRP and NRxRSRP values accurately, enabling more informed handover decisions. By dynamically adjusting the Time-to-Trigger and handover thresholds based on the predicted values, our algorithm achieves a balance between minimizing HOPP events and ensuring reliable handovers.

The dynamic algorithm is superior to the traditional method because we dynamically adjust the threshold through the error of model prediction to adapt to the changing wireless environment, thereby improving handover efficiency and overall system performance. In real scenarios, if the HOPP probability is low, it means that the ping-pong effect occurs less often, thereby improving user experience.

Next, is the issue of limitations. Our study assumes that the RSRP and Near RSRP data collected across different clients are representative of typical network conditions, but potential biases in the dataset, such as uneven data distribution among clients or underrepresented scenarios, could affect the generalizability of our results. Additionally, while Federated Learning offers significant privacy benefits, it introduces computational overhead due to the need for local training on clients and the aggregation of models, which could limit scalability in resource-constrained environments.

Overall, our dynamic handover algorithm provides a robust solution to the challenges of handover management in mobile networks. Future work can build on these findings by exploring advanced machine-learning techniques and expanding the algorithm’s applicability to diverse network environments.

## 6. Conclusions

We combined Federated Long Short-Term Memory (F-LSTM) and our proposed dynamic handover algorithm to reduce unnecessary handovers. The F-LSTM model predicts future Reference Signal Received Power (RSRP) and nearest Reference Signal Received Power (NR-RSRP) values. The predicted NR-RSRP and the Mean Absolute Error (MAE) of the predicted RSRP are utilized as reference values and thresholds for the dynamic handover algorithm, respectively. Compared to the 3GPP A3 Event and Greedy-based handover algorithms, our approach reduced the Handover Ping-Pong Rate (HOPP) by 2.73% and 10.45%, respectively, effectively mitigating the occurrence of the ping-pong effect.

In terms of future research, we suggest exploring the integration of our model with other advanced machine learning techniques, such as reinforcement learning, to further enhance the decision-making process in dynamic network environments. Testing the model in different and more diverse network environments, such as rural or highly congested urban areas, could provide additional insights and validate the robustness of our approach. Finally, optimizing the computational efficiency of the FL framework, possibly through the use of lightweight models or distributed computing techniques, would be a valuable direction for improving scalability and applicability in real-world settings.

## Figures and Tables

**Table 1:** Description of dataset features.

Name	Explanation
Timestamp	Timestamp of sample
Longitude and Latitude	GPS coordinates of mobile device
Velocity	Velocity in kph of mobile device
Operator name	Cellular operator name (anonymized)
CellId	Serving cell for mobile device
NetworkMode	Mobile communication standard (2G/3G/4G/5G)
DL_bitrate	Download rate measured at the device (application layer) (kbps)
UL_bitrate	Uplink rate measured at the device (application layer) (kbps)
State	State of the download process. It has two values, either I (idle, not downloading) or D (downloading)
SNR	Value for signal-to-noise ratio (dB)
RSRP	Value for RSRP. Represents an average power over cell-specific reference symbols carried inside distinct RE. RSRP is used for measuring cell signal strength/coverage, and therefore, cell selection (dBm)
NRxRSRQ & NRxRSRP	RSRQ and RSRP values for the neighboring cell

**Table 2:** Model prediction performance comparison.

	MAE	MSE	RMSE
Hete_FL ^1^	3.5021	5.2394	2.2883
Homo_FL ^2^	3.3728	4.6171	2.1476
Cen_LSTM ^3^	3.0356	18.9824	4.3529

^1^ Heterogeneous federated learning. ^2^ Homogeneous federated learning. ^3^ Centralized LSTM Model.

**Table 3:** Model prediction accuracy in different datasets.

	HSPA+/LTE/5G	LTE/5G	5G
Hete_FL ^1^	0.9531	0.9677	0.9940
Homo_FL ^2^	0.9460	0.9638	0.9901
Cen_LSTM ^3^	0.9458	0.9647	0.9433

^1^ Heterogeneous federated learning. ^2^ Homogeneous federated learning. ^3^ Centralized LSTM Model.

**Table 4:** Comparison of Model Time Costs.

	Training Time (s)	Execution Time (s)	Prediction Time (s)
Hete_FL ^1^	917.630	0.583	0.575
Homo_FL ^2^	134.114	0.336	0.322
Cen_LSTM ^3^	97.572	0.357	0.338

^1^ Heterogeneous federated learning. ^2^ Homogeneous federated learning. ^3^ Centralized LSTM Model.

## Data Availability

No new data were created or analyzed in this study.

## Abbreviations

The following abbreviations are used in this manuscript:

FL	Federated Learning
LSTM	Long Short-Term Memory
F-LSTM	F-LSTM
RSRP	Reference Signal Received Power
HCPs	Handover Control Parameters
TTT	Time-to-Trigger
HOM	Handover Margin
HOPP	Probability of Handover Ping-Pong
ML	Machine Learning
SVM	Support Vector Machines
mmWave	Millimeter-wave
QoS	Quality of Service
NR-RSRP	Nearest Reference Signal Received Power
MAE	Mean Absolute Error
MSE	Mean-Square Error
RMSE	Root-Mean-Square Error
